# Network Analysis of Multivariate Transfer Entropy of Cryptocurrencies in Times of Turbulence

**DOI:** 10.3390/e22070760

**Published:** 2020-07-11

**Authors:** Andrés García-Medina, José B. Hernández C.

**Affiliations:** 1 Unidad Monterrey, Centro de Investigación en Matemáticas, A.C. Av. Alianza Centro 502, PIIT, Apodaca 66628, Nuevo Leon, Mexico; jose.chaudary@cimat.mx; 2 Consejo Nacional de Ciencia y Tecnología, Av. Insurgentes Sur 1582, Col. Crédito Constructor, Ciudad de México 03940, Mexico; 3 Escuela de Matemática, Facultad de Ciencias, Universidad Central de Venezuela, Av. Los Ilustres, Los Chaguaramos, Caracas 1020, Venezuela

**Keywords:** cryptocurrencies, multivariate transfer entropy, complex networks

## Abstract

We investigate the effects of the recent financial turbulence of 2020 on the market of cryptocurrencies taking into account the hourly price and volume of transactions from December 2019 to April 2020. The data were subdivided into time frames and analyzed the directed network generated by the estimation of the multivariate transfer entropy. The approach followed here is based on a greedy algorithm and multiple hypothesis testing. Then, we explored the clustering coefficient and the degree distributions of nodes for each subperiod. It is found the clustering coefficient increases dramatically in March and coincides with the most severe fall of the recent worldwide stock markets crash. Further, the log-likelihood in all cases bent over a power law distribution, with a higher estimated power during the period of major financial contraction. Our results suggest the financial turbulence induce a higher flow of information on the cryptocurrency market in the sense of a higher clustering coefficient and complexity of the network. Hence, the complex properties of the multivariate transfer entropy network may provide early warning signals of increasing systematic risk in turbulence times of the cryptocurrency markets.

## 1. Introduction

Cryptocurrencies are new financial instruments based on the technology of blockchains. A coin is defined as a chain of digital signatures. In Bitcoin, each owner transfers the coin by digitally signing a hash of the previous transaction and the public key of the next owner, adding them to the end of the coin [1]. A cryptocurrency exchange platform is a website on which we can buy and sell coins for other digital currency or trust money. Depending on the exchange, they can operate as a stock exchange or as a currency exchange house, which is very effective and safe for users. The easy access of this market through more than 22,000 projects operating within the industry, exchanges with low fees of transactions, more than 5000 virtual coins worldwide, and a daily trade volume of nearly 174 billion dollars have done cryptocurrencies a very attractive instrument of investment for the general population [2].

The interactions of cryptocurrencies are not always identical among all variables, in other words, the variables influence each other with different magnitude. Thus, it is necessary to study the asymmetric dependence structure to understand such interactions. The common measures to estimate dependencies, i.e., linear cross-correlation, cross-spectra, and mutual information; share the characteristic to be symmetric in nature. The usual approach to understanding asymmetric dependencies is through the parametric approach of copulas [3]. Nevertheless, we are interested in an approach from the point of view of information theory in order to take into account the concept of causality. Even though there exist also attempts to model causality via copula-based methods [4], the information approach works on the broadest sense of free modeling.

The most popular measures of asymmetric dependencies in information theory are related to conditional information or Transfer entropy (TE) based on the concept of Shannon entropy [5]. This quantity was introduced originally with the purpose to quantify the statistical coherence between systems evolving in time [6]. Since then transfer entropy has been used to solve problems of different nature. It has been effective in the study of the neuronal cortex of the brain [7], statistical physics [8], dynamical systems [9], given a thermodynamic interpretation in [10].

In applications to econometrics, transfer entropy can be regarded as a nonlinear generalization of the Granger causality test [11]. There exists a series of results [11,12,13] that state an exact equivalence between the Granger causality and TE statistics for various approaches and assumptions on the data generating processes, which make it possible to construct TE as a non-parametric test of pure Granger causality. In a previous study [14], we compared a synthetic linear and non-linear models, and an empirical data set of cryptocurrencies, where is highlighted the advantage of the symbolic estimation of TE over traditional Granger causality test. Moreover, in [15] the multivariate version of symbolic transfer entropy has been tested, the authors show that it can be applicable to non-stationary time series in mean and variance and is even unaffected by the existence of outliers and vector autoregressive filtering.

Despite this characteristic, the main deficiency of the general TE as a measure of causality is the possibility of spurious causalities due to indirect influences or common drivers. To explain this point consider the processes *X*, *Y*, and *Z*; if a causal interaction is given by X→Y→Z, a bivariate analysis would give a significant link between *X* and *Z* that is detected as being only indirect in a multivariate analysis [16]. An approach to overcome this issue is proposed in [17] by inferring an effective network structure given multivariate interactions using a greedy algorithm. The authors of [18] improved the methodology by adding a preliminary step to prune the set of sources and implementing a series of rigorous statistical tests to decrease the type I and II errors emerged in the multiple comparisons involved on the computations of multivariate transfer entropy. The approach of these studies employs conditional and collective forms of multivariate transfer entropy [19,20].

On the other hand, complex system theory has a long tradition between physicists. The emergence of regularities have been observed in different systems and theories, such as convection, turbulence, phase transition, nonlinear dynamics, renormalization theory, among other fields of physics [21]. However, there is no strict mathematical definition of what complexity is, rather it is characterized according to the properties presented in the particular system. These properties are commonly referred as scale invariance, self-organized criticality, hierarchical structure, coexistence of collective effects and noise, variability and adaptability, and highly optimized tolerance [22].

Network representation has become a common approach to represent interactions between elements in complex systems. This tool allows characterizing the properties of different phenomenan in a common framework. Graph theory has become an essential piece to the understanding of the structure and behavior of these systems. Based on it has been possible to discover emerging properties having fundamental implications on different areas of knowledge [23]. The representation of the financial markets through networks and the study of their complex structure draw attention in physics since the seminal work [24]. There, it is introduced the ultrametric distance and the Minimum Spanning Tree (MST) to characterize the correlations between the stocks used to compute the Dow Jones Industrial Average (DJIA) index, and the portfolio of stocks used to compute the Standard and Poor’s 500 (S&P 500). Also, in [25] it is extended the methodology to study a portfolio of equities at different time horizons, as well as the MST structure of volatilities comparing the network’s topological properties of real and artificial markets.

Subsequently, a series of works have emerged that describes an analogy between the foreign exchange market (or Forex) and the cryptocurrency market, using as a framework the tools that characterize complex networks. To begin with, the work done on [26] studied a large collection of daily time series for world currencies’ exchange rates through MST methodology. They find an autoscaling behavior in the degree distribution of the network, and demonstrate the existence of a hierarchical structure in the currency markets by developing an analytical model. In the simultaneous works [27,28] the authors show that the structure of the Forex network depends on the base currency. In addition, they found the network is not stable in time, noting the USD node gradually loses its centrality, while the EUR node starts turning more central during the study period. In relation to cryptocurrencies, the study [29] analyzes the return distribution, volatility autocorrelation, Hurst exponents, and the effects of multiscaling for the Bitcoin market. There, the authors find that this market show signs of maturity during the last months of the analysis, whose characteristics resemble the complex properties of the Forex market. The analysis done in [30], found further evidence of the shared features of Forex and Cryptocurrency markets at high-frequency rate. Besides, it is pointing out the BTC/ETH and EUR/USD exchange rates do not show any significant relationships. Thus, they hypothesize both markets start decoupling. The same authors extend their study to 100 cryptocurrencies, introducing the collective analysis of random matrices [31]. They found that the level of collectivity depends on which cryptocurrency is used as the exchange rate. Moreover, it is detected that the USD begins to disconnect from the network and resemble a fictitious currency, which may imply the cryptocurrencies’ autonomy. Last, the work of [32] studies the relationship between both markets from a more econometric perspective, where it is explained the importance of diversifying between them.

The previous results pave the way to carry out extensive studies on cryptocurrencies without having to link them to the forex markets, despite the fact both markets share several similarities. In this spirit we are interested to study the complexity properties that emerge in the induced network by multivariate entropy transfer when considering the price and volume of transaction. In this sense, we are seeking to characterize their asymmetric interactions by applying a series of statistical tests with the intention of considering only significant connections. To the aim of bias reduction, we are looking for multivariate rather than bivariate approach. Likewise, it is desired to describe the turbulence observed during March 2020 by interpreting the temporal behavior of the directed interactions via the complex network’s artifacts. In this regard, the turmoil of cryptocurrencies has been analyzed in [33] from the concept of *bull and bear* market. The authors study the three largest cryptocurrencies of Bitcoin, Ethereum, and Litecoin through the technique of Detrended Fluctuation Analysis (DFA) analyzing the Hurst exponent over a different time windows. They find that during the *bull* period the market is efficient, whereas in *bear* times it is inefficient.

Furthermore, there exist literature discussing the spillover effect and systematic risk among the cryptocurrency markets. In [34] it is found that the structural breaks are universally present in seven of the largest cryptocurrencies, whereas it is spreads from the smallest to the largest currencies, in order of capitalization. This finding is done by implementing the Granger causality test, as well as a test for the ARCH and the Dynamic Conditional Correlation MGARCH to the selected coins. Furthermore, the work [35] show evidence that Ethereum is likely to be the independent coin in the cryptocurrency markets, while Bitcoin tends to be the spillover effect recipient. There, the author modeled the system by variants of the Vector Autoregressive Model (VAR), and using jointly distributed Student’s-t copulas to measure the risk contagion among cryptocurrencies. Moreover, the study [36] found a risk contagion effect between cryptocurrencies when applying a copula approach. There, it is suggested to perform portfolio diversification to avoid this phenomenon.

On the perspective of transfer entropy, the study of Sandoval [37] uses the information measure to characterize the contagion of institutions in times of crisis. He identifies the companies most vulnerable to be contagious on countries that have suffered sovereign default. A recent study in similar direction is [38], where transfer entropy is estimated by discretizing the return time series into positive and negative values and validated by resampling. The researchers constructed an indicator to measure the systematic risk on the stock market and real state data. They observed the networks manifest strongly connectivity around periods of high volatility around the crash of 2008. Further, the authors of [39] apply the Rényi Transfer Entropy to investigate the interactions between the crude oil markets and the cryptocurrency markets. Their results suggest that the macroscopic economic value of the US crude oil has an contagion effect on the cryptocurrency markets.

In this study, we analyze the cryptocurrency network induced by the estimation of the multivariate transfer entropy as proposed in [18]. We are especially interested to understand the effects of the financial turbulence of 2020 on the market of cryptocurrencies taking into account the price and volume of transactions as a variable of interest. To obtain deeper insights about the structure of the induced network we quantify the clustering coefficient and estimate the degree distributions of nodes, which are two standard tools from complex networks [40]. Our work follows the line of thought of the literature discussed above, specifically in the sense of studying the systematic risk and contagion between the currencies through the transfer entropy when the cryptocurrency market is in a turbulent situation. Likewise, the March 2020 turmoil is explored with network theory’s tools, which have had a fundamental role in the econophysics interpretation of the complex systems that emerge in finance.

However, our work is distinguished in combining a series of elements that give rise to ask and exploring the following questions, which as far as we know have not yet been discussed in the literature: Do the directed networks associated to the multivariate transfer entropy of volume and price of cryptocurrencies present complex properties? Is it possible to characterize and in some sense to anticipate the turmoils on the cryptocurrency markets through the properties of the network induced by the multivariate transfer entropy? Can the clustering coefficients of these networks play the role of an early indicator of turbulence in these markets? Is there self-similarity in the induced networks, and if so, how do we interpret this characteristic in turbulent times?

We hypothesize the complex properties of the multivariate transfer entropy network may provide early warning signals of increasing systematic risk. This is inferring through evidence found during the turbulence of March 2020 for the induced directed networks by the multivariate information measure of the hourly volume and price of 146 coins from December 2019 to April 2020. We hope, these results may help the practitioners who venture to invest in this risky class of financial instruments to have further quantitative tools to assess systematic risk during times of turbulence.

The next Section 2 describes the data under study. Section 3 presents the greedy algorithm and the series of statistical tests involved in the computation of multivariate transfer entropy, as well as preliminary results. In Section 4 the network dynamics characterized by the clustering coefficient and power law fitness are analyzed in the context of the generated network of cryptocurrencies. Finally, Section 5 highlights the implications of the results and future work is proposed.

## 2. Data

We consider the price and volume in dollars of p=146 cryptocurrencies, which are obtained using the API of CoinMarketCap [2] for the period from 00:00 2019-12-01 to 00:00 2020-04-05, at an hourly frequency, resulting in a total of n=3025 observations (see Supplementary Material). The data acquisition and preprocessing strategy consisted in creating a database on a remote Linux server and automatically make calls to the CoinMarketCap API (in UTC time) to request the price and volume of the first 200 coins in order of capitalization on the day the database was built, i.e., on the 9th November 2019. The frequency and number of currencies were chosen in such a way that it did not exceed the number of requests or credits allowed by the API. The intention was to fulfill the commitment to have the maximum number of coins at the highest possible frequency. The cryptocurrencies are selected under the condition of having each less of 1% of missing values in volume and price during the trading period. In case of no record, a spline interpolation of order three was used to fill the time series gaps. Thus, our set of variables dropped to the reduced variables stated above (p=146). In addition, we transformed the 2p time series into price-returns $r_{k}^{\left(price\right)}\left(t\right)$, and volume-returns $r_{k}^{\left(volume\right)}\left(t\right)$ by computing the logarithmic difference of consecutive observations

(1) $$\begin{array}{cc} r_{k}^{\left(price\right)}\left(t\right) & =\log\left(s_{k}\left(t\right)\right)-\log\left(s_{k}\left(t-1\right)\right) \end{array}$$

(2) $$\begin{array}{cc} r_{k}^{\left(volume\right)}\left(t\right) & =\log\left(v_{k}\left(t\right)\right)-\log\left(v_{k}\left(t-1\right)\right) \end{array}$$

where k=1,…,p; t=1,…,n; and $s_{k}\left(t\right)$, $v_{k}\left(t\right)$ stands for the price and volume of cryptocurrency *k* at time *t*, respectively. In this way, we deal only with the stochastic part of the time series.

Even though the general approximation of the transfer entropy is non-parametric, we are going to use a Gaussian estimator for the conditional distributions as will be explained in the next section. Then, it is necessary to ensure that the data is stationary. For this purpose, it is a usual practice to take as an input the logarithmic returns to satisfy this requirement. Another approach is not to use excessively long time series, even though it may create a compromise between the bias of the transfer entropy estimator and stationarity. In this study, in addition to using moderately long log-returns time series, we have verified stationarity using the augmented Dickey-Fuller test [41] and the Phillips-Perron test [42]. In both tests it has been obtained a *p*-value less than 0.001 for all the 2p time series considered. Therefore, it is confirmed no evidence of a unit root in any variable.

Further, it is known that the distribution of financial and cryptocurrency data change according to the resolution that we observe them [43,44,45]. In this sense, it is important to keep in mind the implications of our results are only valid for hourly observations. Our interest in this frequency lies in the high volume of transactions that take place intra-day on the market of cryptocurrencies. However, we did not want to go to higher frequencies (minutes) due to two situations. The first is that volatility increases and it is more difficult to justify stationarity. Second, the justification for using a Gaussian estimator to calculate the entropy transfer would be invalided because the distribution of the logarithmic returns increases its kurtosis and their distribution start resembles one of the Levy family [45]. On the other hand, we are interested to delimit our work for the period of time just before the pandemic effects take place. Consequently, we have the limitation of having not too many days of transactions around this event, so the sample would not be large enough to obtain an unbiased estimation of multivariate transfer entropy if used daily time series.

Finally, it is important to mention that we are working with non-traded prices. Coinmarketcap is known as a *coin-ranking* service because they rank both coins and exchanges by trading volume and market capitalization to weight their cryptocurrencies data. The specific strategy followed by CoinMarketCap can be found in [46]. This type of information would represent a problem if we were interested in proposing an optimal portfolio, hedging strategies, or trading applications, as is properly warned in [47]. Nonetheless, it is not the goal of this study, we are interested in the weighted information provided by CoinMarketCap, since it estimates the formation of prices considering important features of the cryptocurrency data and selected exchanges. The last is more in tune with the vision of complex systems. In other words, the objective of our study is in essence explanatory rather than predictive. Notwithstanding, it should be kept in mind the results are conditioned to the way in which CoinMarketCap weighted the information.

## 3. Multivariate Transfer Entropy

The Transfer Entropy (TE) from a process *X* to a process *Y* measures the amount of uncertainty reduced in future values of X by knowing the past states of Y and X itself. In other words, transfer Entropy (TE) quantifies the amount of information that the past of a source process *X* provides about the current value of a target process $Y=y_{t}$, considering the context of *Y*’s own past. In a multivariate setting, a set of sources $X_{i},i=1,\ldots ,M$ is provided. The multivariate Transfer Entropy (mTE) from $X_{i}$ to *Y* can be defined as the information that the past of $X_{i}$ provides about $Y=y_{t}$, in the context of both *Y*’s past and all the other relevant sources in *X*. The main challenge is to define and identify the relevant sources. In principle, the mTE from $X_{i}$ to *Y* is computed by conditioning on all the other sources in the network, i.e., $X\setminus X_{i}$. However, in practice, the sizes of the conditioning set must be reduced in order to avoid the curse of dimensionality. The idea is to restrict the conditioning set by finding and including the sources that participate with $X_{i}$ in reducing the uncertainty about $Y=y_{t}$, in the context of *Y*’s own past. The set of relevant sources will be denoted as *Z* [48].

In order to infer *Z* from *X* it is followed the greedy algorithm approach suggested by [17,18], where *Z* is built iteratively by maximizing the conditional mutual information (CMI) criterion. As a first step, a set of candidate variables c∈C is defined from the past values of *X*; then, *Z* is built including iteratively the candidate variables *c* that provides statistically significant maximum information about the current value $Y=y_{t}$, conditioned on all the variables that have already been selected. More formally, at each iteration, the algorithm selects the past variable c∈C that maximizes the conditional mutual information $I(c,y_{t}|Z)$ at significance level α of a maximum information test. The set of selected variables forms a multivariate, *non-uniform embedding* of *X* [49] (See Appendix A).

The implemented greedy algorithm operates in several steps. First, *Z* is initialized as an empty set and is considered the candidate sets for *Y*’s past $C_{Y}$ and *X*’s past $C_{X}$. Second, variables from $C_{Y}$ are selected. To this end, for each candidate, $c\in C_{Y}$, it is estimated the information contribution to $Y=y_{t}$ as $I(c,y_{t}|Z)$. Next, it is found the candidate with maximum information contribution, $c^{*}$, and is performed a significance test; if it is found significant, must be added $c^{*}$ to *Z* and remove it from $C_{Y}$. In addition, the *maximum statistic* (see Appendix B.1) is used to test for significance while controlling the Family-Wise Error Rate (FWER). Then, the algorithm stops if $I(c^{*},y_{t}|Z)$ is not significant or $C_{Y}$ is empty. Third, variables are selected from *X*’s past states, i.e., iteratively candidates in $C_{X}$ are tested following the procedure described before. Fourth, redundant variables in *Z* are tested and removed using the *minimum statistic* (see Appendix B.2). The minimum statistic computes the conditional mutual information between every selected variable in *Z* and the current value, conditional on all the remaining variables in *Z*. This test is performed to ensure that the variables included in the early steps of the iteration still provide a significant information contribution in the context of the final parent set *Z* [17]. Fifth, the *omnibus test* is performed (see Appendix B.3) to test the collective information transfer from all the relevant source variables to the target $I(Z_{X},y_{t}|Z_{Y})$. The resulting omnibus *p*-value is later used for the correction of the FWER at the network level. If the omnibus test is significant, a sequential maximum statistic is performed on each selected variable z∈Z to obtain the final information contribution and *p*-value for each variable.

The mTE between a single source $X_{i}$ and target *Y* can be computed from the inferred non-uniform embedding *Z*. To this end, it is collected from *Z* all of $X_{i}$’s selected past variables, $X_{i}$, and calculated the mTE as $I(X_{i};y_{t}|Z\setminus X_{i})$. Note that time lag between $X_{i}$’s selected past variables and the current value at time *t* indicates the information-transfer delay [50]. The delay can be estimated as the lag of the past variable which provides the maximum individual information contribution, where the maximum contribution is indicated either by the maximum raw TE estimate or by the minimum *p*-value over all variables from the source. Finally, the algorithm must be repeated for every node (or variable) in the network (see [18] for an extensive description of the mTE algorithm and hypothesis testing).

### mTE Network of Cryptocurrencies

We consider each time series of price-returns and volume-returns of cryptocurrencies to be a stochastic process in order to detect the causal relations between the variables. The procedure is to fix the target variable $Y=X_{i}$, the source set as $X\setminus X_{i}$, and apply the mTE algorithm [48] described above for each time series i=1,…,2p, where the first *p* variables represent the price-returns and the last *p* variables the volume-returns, where p=146 as described in the data section. The intention is to detect if the causal relations of price-returns and volume-returns bring clues to understand the dynamic of cryptocurrency market in times of turbulence. Hence, we design a temporal analysis of time windows of 21 days, sliding by seven days, and using an overlapping of 14 days. Under this procedure, it is obtained k=16 time windows, the first from the 01:00 of 2019-12-01 to 00:00 of 2019-12-22, and the last from 01:00 of 2020-03-15 to 00:00 of 2020-04-05. Thus, each data frame contain q=504 observations of hourly price-returns and volume-returns, i.e., having dimensions q×2p.

It is of primary importance to estimate the CMI, represented above as $I(X_{i},y_{t}|Z\setminus X_{i})$, in order to quantify mTE, where i=1,…,2p. We estimate CMI under the assumption that price-returns and volume-returns follow a jointly Gaussian continuous distribution, which is equivalent to assume a linear causal dependency as proposed by Granger [51]. The value added to the original Granger causality test is the use of a multivariate framework to test statistically significant causal relations conditioned to other variables, while the original test only measures bivariate dependencies. We chose Gaussian estimator over a more realistic distribution approach due to the dimension settings as well as the sliding windows turn the computational complexity excessively time demanding. Nevertheless, the results assuming linear dependencies are worthwhile to mention due to the capacity of mTE to detect conditional interactions.

**Figure 1:**
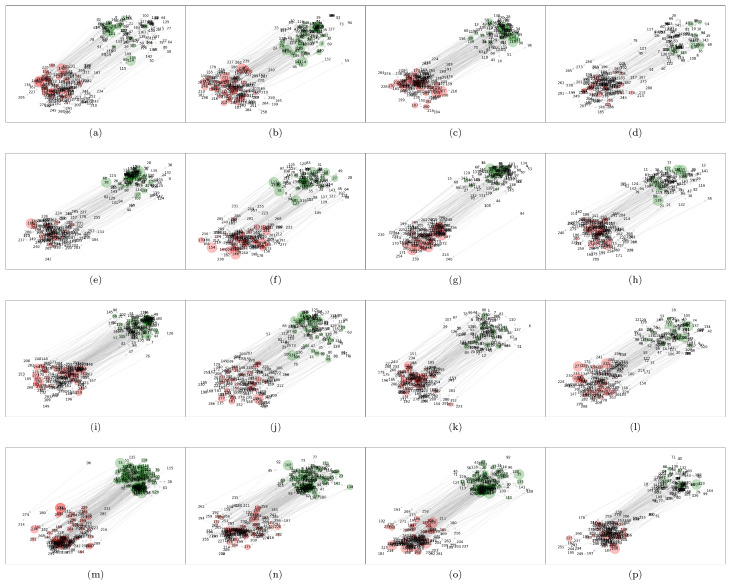
Network representation of mTE results for cryptocurrency variables. The green nodes represent price-return variables, while the red nodes represent volume-return variables. The subfigures (**a**–**p**) show the directed network results for the time window under study. The subfigures are arranged in temporal order from top-left to right-bottom.

Figure 1 shows the resulting networks for the mTE analysis of cryptocurrencies described above using the Gaussian estimator. Here we set the number of permutations for the surrogate distribution used in the statistical tests (*maximum*, *minimum*, and *omnibus*) to 500, and a significance level of 0.05. The alphabetic order of the subfigures represents the temporal order of the time window of each experiment. Thus, for example, the subfigure (a) shows the results for the time frame 2019-12-01–2019-12-22, the subfigure (b) for 2019-12-08–2019-12-29, and so on. The graph visualization is made using the Kamada-Kawai algorithm [52], where each variable is represented as a node. We have discriminated the price and volume variables separating their corresponding nodes by a fixed distance to the upper right if it corresponds to a price-return node, and to the lower left if it corresponds to a volume-return node. In addition, the price nodes are colored in green, while the volume ones in red. The directed edges represent true causal relations under the mTE methodology, where we have tested for one to three lags, and as a result, it is obtained a binary adjacency matrix *A*. This matrix has elements $A_{ij}=1$ if there exit a statistically significant causal relation in this range of lags, and $A_{ij}=0$ on the contrary, where i,j=1,…,2p. Finally, the size of each node is drawn according to its clustering coefficient, which is described in the next section. However, here it is already noting the increasing size of the green nodes for the cases (m)–(o), which correspond to the subnetwork of price-returns for the time windows ending at 2020-03-15, 2020-03-22, and 2020-03-29, respectively.

## 4. Complexity Behavior

In this section, we describe and quantify for the data sets under study two essential quantities in complex network theory: clustering coefficient and degree distribution. The former in order to measure cliques or connectivity and the latter to estimate the scale-free property of the network.

### 4.1. Clustering Coefficient

The tendency of a network to form tightly connected neighborhoods, more than in the random uncorrelated case, can be measured by the clustering coefficient (CC) [53]. Consider a network described by the graph G=(V,A), where *V* is the number of nodes, and $A=\{a_{ij}\}$ is the V×V adjacency matrix. The orientation of edges or arrows in directed networks turn the adjacency matrix to be non-symmetrical in general. In a binary directed networks, as are the induced networks by mTE, the node out-degree $k_{i}^{out}$ is the number of edges pointing out from the node *i*, while the node in-degree $k_{i}^{in}$ is the number of edges pointing towards the node *i*. Formally

(3) $$\begin{array}{cc} k_{i}^{out} & =\sum_{j\neq i}a_{ij}={\left(A\right)}_{i}1 \end{array}$$

(4) $$\begin{array}{cc} k_{i}^{in} & =\sum_{j\neq i}a_{ji}={\left(A^{\prime }\right)}_{i}1, \end{array}$$

where $A^{\prime }$ is the transpose of *A*, ${\left(A\right)}_{i}$ is the *i*th row of *A*, and 1 represents the column vector ${\left(1,1,\ldots ,1\right)}^{\prime }$ of dimension *V*. Similarly, the total degree $k_{i}^{tot}$ of node *i* is the sum of its in-degree and out-degree

(5) $$k_{i}^{tot}=k_{i}^{in}+k_{i}^{out}={\left(A^{\prime }+A\right)}_{i}1.$$

Further, assuming that no self-interactions are present, the bilateral edges $k_{i}^{↔}$ between *i* and its neighbors are counted as

(6) $$k_{i}^{↔}=\sum_{j\neq i}a_{ij}a_{ji}=A_{ii}^{2}.$$

The usual approach to quantify the clustering coefficient of node *i* is by measuring the ratio of the number of triangles in the graph *G* with *i* as one vertex over the number of all possible triangles that *i* could form. In the directed graph case, the clustering coefficient $C_{i}$ for node *i* can be explicitly computed by the expression [54]

(7) $$C_{i}=\frac{{\left(A+A^{\prime }\right)}_{ii}^{3}}{2[k_{i}^{tot}\left(k_{i}^{tot}-1\right)-2k_{i}^{↔}]},$$

and the overall clustering coefficient *C* for the directed graph *G* is obtained by $C=V^{-1}\sum_{i=1}^{V}C_{i}$, where C∈[0,1].

**Figure 2:**
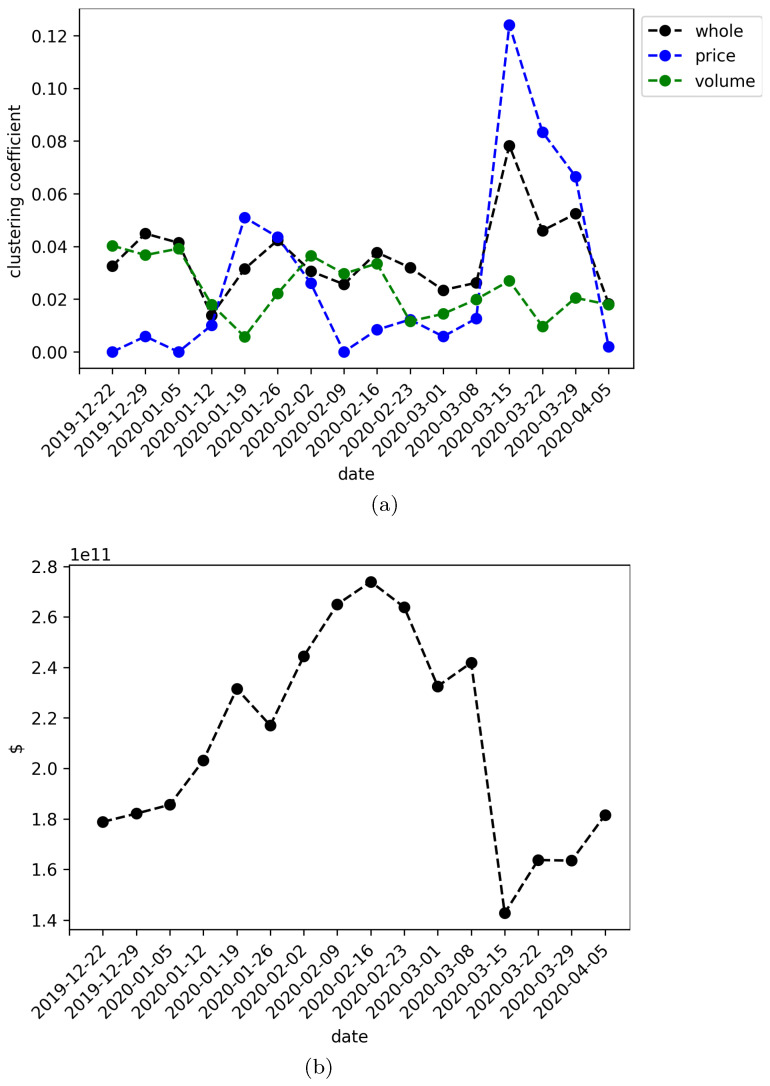
(**a**) Overall clustering coefficient as a function of time. Black dotted lines show the results for the whole networks, blue and green dotted lines show results for the price-return and volume-return nodes, respectively. (**b**) Market capitalization averaged over the cryptocurrencies under study at the end of the time window. The units in the y-axis are measured in hundred million dollars.

Figure 2a shows the overall clustering coefficient as a function of time for the networks associated with each time window of the data set. The black dotted line are the results for the whole networks (i.e., volume-return and price-return nodes), whereas the blue and green dotted lines represent the results for the price-return and volume-return nodes, respectively. It is remarkable the peak in the clustering coefficient for the time window ending at 2020-03-15, especially in the price-return subnetwork and in less amount for the whole network.

In order to gain insight that leads us to a better understanding of this behavior, it is superposed the market capitalization at the end of the time window of each experiment. Thus, Figure 2b shows the average market capitalization in dollars, taking into account the observed values of each cryptocurrency of the data set. It is worth noting the coincidence of the fall in the average of market capitalization with the increase of the overall clustering coefficient for the whole and price-returns networks at date 2020-03-15. This abrupt change in both behaviors is situated at the period of most severe contraction of the global stock markets due to the recent COVID-19 worldwide pandemic. These results suggest that the financial turbulence induce a higher flow of information to the cryptocurrency market on its price-returns features and as a consequence the overall clustering coefficient increase. In the next subsection is analyzed the degree distribution of the networks with the intention to sustain this hypothesis from another angle.

### 4.2. Power Law

Power laws are probability distributions with the form $p\left(x\right)\propto x^{-\alpha }$, which have the characteristic of being heavy-tailed. This feature may be so extreme that standard deviation only can be defined if α<3, or the mean if α<2. Phenomena following a power law distribution are known as scale-free systems because all values are expected to occur, without a characteristic size or scale. These kinds of distributions have been identified throughout nature, including physics and finance [44,55,56,57] to cite some relevant examples in the context of this work.

**Figure 3:**
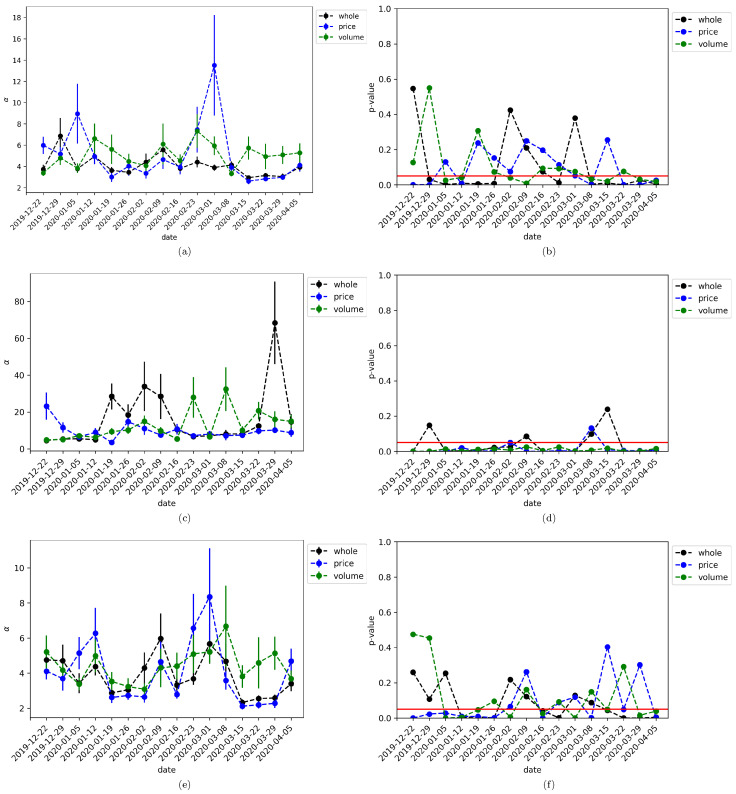
The Behavior of the mTE network structure through time. (**a**,**b**) show the dynamic of the estimated power α and corresponding *p*-value for the degree distribution. (**c**,**d**) show the dynamic of the estimated power α and corresponding *p*-value for the in-degree distribution. (**e**,**f**) show the dynamic of the estimated power α and corresponding *p*-value for the out-degree distribution. In all cases, the black line represents the results for the whole network, the blue line for the price-returns subgraph, and the green line for the volume-returns subgraph. The red straight line is the significance threshold of 0.05 and the vertical lines centered at each point represents the standard deviations.

We use the powerlaw library [58] to fit a power law to a degree, in-degree, and out-degree distributions for the induced graph by the mTE flow of information of each time window under study. The results are shown in Figure 3 for the whole network, as well as the price-returns and volume-returns subgraphs, where the vertical lines represent the standard deviation of the adjustments. Also, it is plotted the *p*-value of fitness using the log-likelihood ratio test, wherein all cases the null hypothesis assumes the distributions are characterized by an exponential function. Table 1, Table 2 and Table 3 show the numerical results associated to Figure 3.

Let analyze first the degree distribution case. As can be seen from Figure 3a there is a peak at 2020-03-01 for the price-returns subgraph, where the estimated fit of the degree distribution is α=13.5086, whereas the whole and volume-returns degree distribution is adjusted with a power oscillating below eight. Figure 3b tells us that the adjustment is not significant most of the time, where we can see *p-values* over the threshold of 0.05 (red line). Nevertheless, the peak at 2020-03-01 for the price-returns is at the border of significant fitness. In a similar way, Figure 3c shows the estimated power behavior for the in-degree distribution, where now there exists an abrupt change at 2020-03-29 for the whole network with an estimated α=68.3999. In this scenario, the adjustment is significant most of the time as can be seen in Figure 3d. Finally, Figure 3e,f shows the corresponding results for the out-degree distribution, being all the estimated power below nine, but without a significant pattern.

**Figure 4:**
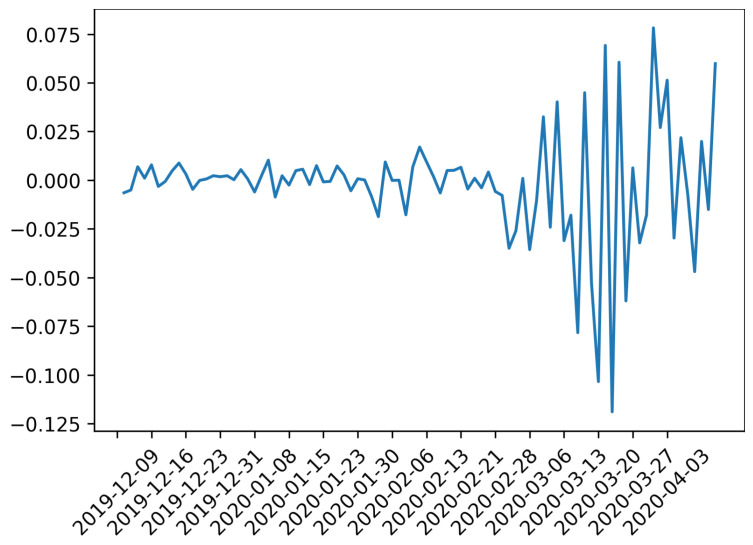
Daily log-returns of ACWI for the trading dates from 2019-12-02 to 2020-04-06.

In short, these results confirm what was found in the previous subsection, in the sense that the change in the estimated power of the degree distribution of the price-returns anticipates the fall of the average market capitalization of cryptocurrencies occurring at 2020-03-15. Particularly, the in-degree distribution shows the most robust results in the sense of statistically significant evidence of a peak at end of March for the whole network. Then, it is a better indicator of the complex properties of the mTE network. It is important to keep in mind that during March the global markets become extremely turbulent because of the COVID-19 pandemic and the subsequent crash of oil prices around the world. As a matter of fact, during the period of time under study the All Country World Index (ACWI) fell around 10% on a single day, which is its largest decline since 2008. This index published by Morgan Stanley Capital International or MSCI inc. is a market capitalization-weighted index designed to provide a broad measure of equity-market performance throughout the world taking into account stocks from 23 developed countries and 24 emerging markets [59]. As such it is an adequate thermometer of the world financial situation. In Figure 4 it is shown the daily log-returns for the time window from 2019-12-02 to 2020-04-06 (data are taken from [60]). There can be seen the financial turbulence start at the end of February and reach its highest volatility at the middle of March, which coincide with the lowest point of the market capitalization of Figure 2b Hence, based on the evidence found in this study, it is argued that periods of economic contractions are preceded by a high power law distribution of node degrees for price-returns. Nevertheless, it is necessary to develop extensive studies between global financial indices and cryptocurrencies to properly justify this hypothesis.

## 5. Discussion

The multivariate approach of transfer entropy followed here is an efficient greedy methodology based on multiple hypothesis testing able to detect true causal interactions. Even though the Gaussian specification for the conditional distributions involved in the computations of mTE do not capture non-linear structures in the data, the induced networks of cryptocurrencies exhibit interesting characteristics. Actually, the graph visualization of the interactions of price-returns and volume-returns using the Kamada-Kawai algorithm already displays an informative picture. Under this descriptive representation has been possible to distinguish three time windows where the strength of the attractive forces between nodes stand out to be stronger than the other scenarios. Interestingly, these periods coincide with the most severe fall of the recent worldwide stock markets crash on March 2020.

The clustering coefficient version for directed graphs was also measured. In this case, we first notice that the individual coefficient also increases during the times mentioned above, which motivated us to analyze the temporal behavior of the clustering coefficient for the whole network. Here, our greatest contribution arose finding that the clustering coefficient of the whole network, as well as the price-returns subnetwork, increases dramatically during the same periods of major financial contraction, where we use as an indicator of turbulence the market capitalization of the cryptocurrencies under study.

In addition, we explored deeper the structure of the network through the analysis of the degree distribution of nodes as well as in-nodes and out-nodes. Our intention was to characterize the complexity of the network estimating the power of the associated distributions. Although several of the estimations were not significant, the log-likelihood in all cases bent over a power law distribution, giving evidence of the complex nature of the network. Most importantly, it was found that the power of the distribution has higher estimated values during March 2020, which provides further support to our hypothesis: the structure of the induced cryptocurrency network by mTE changes during times of turbulence in the sense of higher clustering coefficient and complexity.

Future work involves the use of an extensive data set to include the market capitalization, financial indices, sentiment indicators of textual data, as well as volume in a cleaver way in order to verify if the same phenomena are presented in the induced graphs by a more complete data set. The last because what is found here volume does not play a role as relevant as the price is to early-warning signals for future markets turbulence. A forthcoming work is analyzing the same data set from data mining techniques as is the point of view of association rules and the apriori algorithm, where preliminary results already show a rich network structure.

Finally, it is important to clarify that despite the statistical results found here in relation to the power fitness of degree distributions, no economical theory behind power laws has been properly developed yet. At most, we can say the power law degree distributions as well as the clustering coefficient of mTE networks may serve an early warning signal of an increasing systematic risk of the cryptocurrency markets in times of crash. The econophysics community has been put a lot of effort to unravel stylized facts of the network structure of financial markets in general, but it is still necessary to build epistemologically the blocks of the theory jointly with the financial economists to have a common playground to discuss and construct new ideas. As stated in [61]: “*the time is ripe for economists to use those power laws to investigate old and new regularities with renewed models and data*”. In this sense, this work only tries to contribute with new evidence in the networks induced by mTE, hoping to soon have an interpretable theory in the same sense as phase transitions, critical points, and scale invariance of turbulent dynamics are in physical statistics. 

## Figures and Tables

**Table 1:** Degree distribution estimation.

Date	α (Whole)	*p*-Value	α (Price)	*p*-Value	α (Volume)	*p*-Value
2019-12-22	3.738	0.5464	5.9778	0.0006	3.3814	0.1268
2019-12-29	6.8615	0.0314	5.1602	0.0006	4.7913	0.5496
2020-01-05	3.7785	0.0011	8.9531	0.1303	3.8275	0.026
2020-01-12	4.9396	0.0085	4.9325	0.0057	6.619	0.0423
2020-01-19	3.6251	0.0057	3.0092	0.2377	5.603	0.3073
2020-01-26	3.4424	0.0081	4.031	0.1531	4.4695	0.0732
2020-02-02	4.4084	0.424	3.3453	0.075	4.0688	0.0401
2020-02-09	5.5648	0.2106	4.6502	0.2502	6.0909	0.0094
2020-02-16	3.8022	0.0759	3.9331	0.1972	4.5331	0.0938
2020-02-23	4.4157	0.0128	7.455	0.1146	7.3266	0.0924
2020-03-01	3.8864	0.3795	13.5086	0.052	5.9385	0.0754
2020-03-08	4.1438	0.0036	3.9098	0.0005	3.3258	0.0334
2020-03-15	2.9365	0.0096	2.6145	0.2548	5.7256	0.0212
2020-03-22	3.1472	0	2.8274	0.0004	4.9333	0.0764
2020-03-29	3.0356	0.0267	2.9792	0.0006	5.0786	0.0313
2020-04-05	3.9247	0.0041	4.1092	0.0255	5.2757	0.0193

**Table 2:** In-degree distribution estimation.

Date	α (Whole)	*p*-Value	α (Price)	*p*-Value	α (Volume)	*p*-Value
2019-12-22	4.5806	0.0002	23.1967	0.0001	4.8769	0
2019-12-29	5.2993	0.1479	11.592	0.0001	5.076	0.0002
2020-01-05	5.4633	0.0009	6.4081	0	7.1131	0.0132
2020-01-12	4.9525	0.0029	8.9783	0.0203	6.1964	0.0015
2020-01-19	28.4241	0.0029	3.4932	0	9.3489	0.0117
2020-01-26	18.3479	0.0222	14.7033	0.0042	10.2065	0.0141
2020-02-02	33.9089	0.025	11.0187	0.0498	14.9042	0.0068
2020-02-09	28.4241	0.0855	7.5029	0	9.7462	0.0251
2020-02-16	10.5242	0.0002	10.6918	0.0003	5.3289	0.0042
2020-02-23	6.695	0.0025	7.1155	0.003	27.8885	0.0243
2020-03-01	6.9885	0.0015	8.0237	0.0018	6.5015	0.0014
2020-03-08	8.0302	0.0981	7.1173	0.1321	32.3699	0.0056
2020-03-15	8.135	0.2385	7.4099	0.0083	10.02	0.0182
2020-03-22	12.3518	0.0001	9.7665	0.0045	20.6977	0.0003
2020-03-29	68.3999	0.0002	10.1634	0.0035	16.028	0.0014
2020-04-05	14.6396	0.0042	8.732	0.007	14.9042	0.0155

**Table 3:** Out-degree distribution estimation.

Date	α (Whole)	*p*-Value	α (Price)	*p*-Value	α (Volume)	*p*-Value
2019-12-22	4.7432	0.2594	4.1039	0.0001	5.208	0.4752
2019-12-29	4.698	0.1075	3.6869	0.0229	4.1616	0.4543
2020-01-05	3.4208	0.2542	5.1354	0.0276	3.3738	0.0002
2020-01-12	4.3711	0.0037	6.265	0.009	4.979	0.004
2020-01-19	2.8673	0.0064	2.6074	0.009	3.5196	0.0464
2020-01-26	3.0402	0	2.7225	0.0011	3.2296	0.0955
2020-02-02	4.2886	0.2173	2.6393	0.0657	3.0792	0.0057
2020-02-09	5.9639	0.1216	4.6401	0.2623	4.2951	0.1614
2020-02-16	3.3214	0.0411	2.7803	0.0019	4.3985	0.0263
2020-02-23	3.6758	0.0011	6.5573	0.089	5.0874	0.0921
2020-03-01	5.6642	0.1289	8.3496	0.1186	5.2096	0
2020-03-08	4.6742	0.0873	3.5777	0.0019	6.6722	0.1489
2020-03-15	2.3148	0.0437	2.1134	0.4035	3.8052	0.0488
2020-03-22	2.542	0	2.1833	0.0486	4.5843	0.2916
2020-03-29	2.5827	0.0003	2.2731	0.3013	5.127	0.0152
2020-04-05	3.3965	0.0045	4.6771	0.0022	3.6785	0.0392

## Supplementary Materials

The following are available online at https://www.mdpi.com/1099-4300/22/7/760/s1, File S1: Cryptocurrency prices, File S2: Cryptocurrency volumes, Table S3: Cryptocurrency tickers.

## Appendix A. Non-Uniform Embedding

In order to describe the dynamics of the processes, it is necessary to define composite processes consisting of embedding vectors. Let denote as $x_{i,t}$ the state visited by $X_{i}$ at time *t*, where $X_{i}$ can be characterized by the *k* past states of the processes $X_{i,t}^{k}=\left(x_{i,t-1},\ldots ,x_{i,t-k}\right)$. In the classical uniform embedding framework, the embedding vectors are

(A1) $$\begin{array}{c} X^{\left(K_{j}\right)}=\left(X_{1,t}^{\left(k_{1}\right)},\ldots ,X_{j-1,t}^{\left(k_{j-1}\right)},X_{j+1,t}^{\left(k_{j+1}\right)},\ldots ,X_{M,t}^{\left(k_{M}\right)}\right) \end{array}$$

(A2) $$\begin{array}{c} X^{\left(K\right)}=\left(X_{1,t}^{\left(k_{1}\right)},\ldots ,X_{M,t}^{\left(k_{M}\right)}\right). \end{array}$$

These vectors are used to calculate the conditional entropies involved in the estimation of transfer entropy. The basis of a non-uniform embedding relies on not imposing a priori the form of the embedding vectors. Thus, its shape is determined sequentially by selecting in a progressive manner the terms that contribute most to the explanation of the observed dynamics. These terms are taken from a candidate set, which includes the past states of the processes, $X_{1},\ldots ,X_{M}$. To quantify the causality from $X_{j}$ to $X_{i}$ it is necessary to contrast the entropy of $X_{i}$ measured after conditioning on the past of all processes, and after conditioning on the past of all processes except $X_{j}$. To accomplish this, it is defined two different sets of initial candidate terms. The first include the past states of $X_{j}:\Omega =\left\{x_{i,t-l}|i=1,\ldots ,M;l=1,\ldots ,L\right\}$; and the second exclude the past states of $X_{j}:\Omega^{-j}=\left\{x_{i,t-l}|i=1,\ldots ,M,i\neq j;l=1\ldots ,L\right\}$, where *L* is the maximum lag at which the past of each process is investigated [49].

## Appendix B. Statistical Tests

### Appendix B.1. Maximum Statistics

Assume that has been chosen the candidate variable $C^{*}$ from the candidate target past set $Y_{<t}^{C}=\left\{y_{t-1},\ldots ,y_{t-L}\right\}$, which maximizes the CMI contribution. The maximum statistic test reflects this selection process by choosing the maximum value among the surrogates time series. Explicitly, let $I^{*}:=I\left(C^{*},y_{t}|Y_{<t}^{S}\right)$ be the maximum contribution. To test $I^{*}$ for statistical significance the next algorithm is used t [18]:

1. Generate *S* surrogates time series $C_{j,1}^{\prime },\ldots ,C_{j,S}^{\prime }$ for each $C_{j}\in Y_{<t}^{C}$, and calculate the corresponding surrogate CMI values $I_{j,1}^{\prime }=I\left(C_{j,1}^{\prime },y_{t}|Y_{<t}^{S}\right),\ldots ,I_{j,S}^{\prime }=I\left(C_{j,S}^{\prime },y_{t}|Y_{<t}^{S}\right)$. The number of surrogates *S* are chosen in line to the desired significance level $\alpha_{max}$.
2. For each surrogate s=1,…,S, calculate the maximum CMI value over the candidates $I_{s}^{*}:=max\left(I_{1,s}^{\prime },\ldots ,I_{n,s}^{\prime }\right)$, where *n* denotes the number of candidates (the number of comparisons). The values $I_{1}^{*},\ldots ,I_{S}^{*}$ form the empirical null distribution of the maximum statistic.
3. Compute the *p*-value for $I^{*}$ as the proportion of values from the surrogate maximum statistic that are larger than $I^{*}$.
4. Finally, $I^{*}$ is considered significant if the *p*-value is smaller than $\alpha_{max}$.

### Appendix B.2. Minimum Statistics

The minimum statistic test is used to remove the selected variables that have become redundant in the context of the final set of selected source past variables $X_{<t}^{S}$. Also, it controls the FWER due to the multiple comparisons involved in the pruning procedure. If we replace *maximum* with *minimum* in the algorithm described above, then it is obtained the algorithm for the minimum statistic test [18].

### Appendix B.3. Omnibus Test

Let $T^{*}:=I\left(X_{<t}^{S},y_{t}|Y_{<t}^{S}\right)$ be the collective transfer entropy from the selected sources past variables $X_{<t}^{S}$ to the target variable $y_{t}$. It is necessary to test $T^{*}$ for statistical significance against the null hypothesis of zero transfer entropy. Thus, using a similar procedure as described in the section of *maximum statistic* test, the null distribution is obtained via shuffling the realizations of the selected sources. The idea behind is to find the set of relevant sources for each node testing all the selected sources collectively [18].
